# Review of the phenotypes and genotypes of Bardet-Biedl syndrome from China

**DOI:** 10.3389/fgene.2023.1247557

**Published:** 2023-11-15

**Authors:** Zou Xin-Yi, Dai Yang-Li, Zeng Ling-Hui

**Affiliations:** ^1^ Department of Clinical Medicine, Medical School of Hangzhou City University, Hangzhou, China; ^2^ Department of Endocrinology, The Children’s Hospital of Zhejiang University School of Medicine, Hangzhou, China

**Keywords:** Bardet-Biedl syndrome, cilia dysfunction, genotype, phenotype, diagnosis, China

## Abstract

**Objective:** To analyze the phenotypes, genotypes, and the relationship of phenotypes and genotypes for Chinese patients with Bardet-Biedl syndrome (BBS).

**Methods:** The Chinese Wanfang and Weipu data, and PubMed were searched up to December 2022. Patients with detailed clinical feature data were involved in the analysis.

**Results:** A total of 153 Chinese patients, including 87 males, 53 females, and 12 unknown, were enrolled. Their ages ranged from 1.2 to 44 years old with a mean of 16.70 ± 9.90 years old. Among these patients, 80 (52.29%) were reported by ophthalmologists, and only 24 (15.68%) reported by pediatricians. Most patients (132/137, 96.35%) had visual problems; 131/153 (85.62%) had polydactyly; 124/132 (93.93%) were overweight or obese; 63/114 (55.26%) had renal abnormalities; kidney dysfunction was found in 33 (21.57%); 83/104 (79.81%) had hypogonadism and/or genital hypoplasia; and 111/136 (81.62%) had mental retardation. In this series, genetic analysis was performed in 90 (58.82%) patients, including 22 *BBS7* (24.71%), 20 *BBS2* (22.73%), and 10 *BBS10* (11.24%) patients. Moreover, 11 fetuses were diagnosed prenatally in the last 4 years except for one patient in 2004 year. It was noted that *BBS7* had higher penetrance. *BBS2* had higher hearing impairment and lower renal abnormality penetrance. *BBS10* also had lower renal abnormality penetrance as well.

**Conclusion:** Misdiagnosis or miss diagnosis of BBS may be common in China. In patients with polydactyly, visual impairment, obesity, renal abnormalities, hypogonadism, and mental retardation, or in fetuses with polydactyly and/or renal abnormalities, BBS should be considered in the differential diagnosis. Other deformities should be evaluated carefully and genetic analysis should be performed as early as possible.

## 1 Introduction

Bardet-Biedl syndrome (BBS, OMIM 209900) is an autosomal recessive disease, caused by a variant of BBS-related genes. It was first described in 1920 by Dr. Georges Bardet. It is a non-motorized ciliary dysfunction that affects multiple systems. The prevalence of BBS is about 1/125,000∼1/160,000 with regional and ethnic differences (Moore et al., 2005; Tsang et al., 2018). A high incidence (around 1/13,500 newborns) among Arab Bedouins in the Arabian Peninsula, North African desert regions, and Newfoundland’s high blood isolation population is related to consanguineous marriage (Farag and Teebi, 1988; Farag and Teebi, 1989; Gouronc et al., 2020). It is characterized by retinitis pigmentosa, obesity, renal abnormalities, polydactyly, mental retardation, and hypogonadism. Other symptoms including ophthalmic diseases (e.g., astigmatism, strabismus, cataracts), hearing impairment, craniofacial congenital anomalies, hypoplastic teeth, toe deformity, short stature, asthma, neurological impairment (e.g., ataxia, hyperspasmia, olfactory abnormalities, behavioral and mental disorders, and poor coordination), digestive system abnormalities (e.g., intestinal atresia, imperforate anus, congenital megacolon, liver fibrillation), metabolic abnormalities (e.g., insulin resistance, hyperglycemia, dyslipidemia, hypothyroidism, hypertension), and cardiovascular abnormalities, have also been reported (Mujahid et al., 2018; Tsang et al., 2018; Guardiola et al., 2021; Zhong et al., 2023).

BBS is a ciliopathy and has been shown to be closely related to dysfunction of immotile cilia. To date, 28 genes have been reported to be associated with the BBS phenotypes, including 2 candidate genes (*SCLT1* and *SCAPER*) and 2 contributors (*NPHP1* and *TTC21B*) (Niederlova et al., 2019; Khan et al., 2023; Gnanasekaran et al., 2023). In comparison, pathogenic variants in the BBS genes involved in encoding the BBSome complex, including *BBS1* (OMIM 209901), *BBS2* (OMIM 606151), *BBS4* (OMIM 600374), *BBS5* (OMIM 603650), *BBS7* (OMIM 607590), *BBS8/*TTC8 (OMIM 615985), and *BBS9/PTHB1* (OMIM 607968), were the most common, followed by BBS genes involved in encoding BBSome complex “chaperone-like” proteins, including *BBS6/*MKKS (OMIM 604896), *BBS10* (OMIM 610148)*,* and *BBS12* (OMIM 610683) (Seongjin et al., 2009; Dai et al., 2022; Melluso et al., 2023). BBS proteins are needed for the maintenance of ciliary structure and function. The BBSome complex is an essential component of cilia differentiation. It mediates the transport of proteins to the membrane structure of cilia and participates in their structure formation and function (Seongjin et al., 2009). BBSome complex chaperone-like proteins are involved in the regulation of the BBSome complex, which together with members of the Rab family of proteins to promote intraflagellar transport (Yan and Shen, 2022; Melluso et al., 2023). Molecular diagnosis can be clarified by genetic testing in about 80% of patients. Most studies reported that *BBS1* and *BBS10* are the most frequently implicated genes (Mujahid et al., 2018). However, still about 20% of patients currently do not have a definitive molecular diagnosis currently (Forsythe et al., 2018), presenting a significant challenge for diagnosis and genetic counseling.

We aim to highlight the genotype-phenotype relationship and the diagnosis of BBS by summering the BBS patients reported from China. Studies in Chinese and English from China were all reviewed.

## 2 Materials and methods

### 2.1 Data collection

According to the PRISMA guidelines, we reviewed the Wanfang and Weipu data in Chinese using “Bardet-Biedl syndrome”, “Laurence-Moon-Biedl syndrome”, “Laurence-Moon-Bardet-Biedl syndrome”, or “Polydactyly-Obesity-Renal-Ocular syndrome” and in Chinese or English, also above keyword and “China or Chinese” in PubMed up to December 2022. Only patients with detailed clinic feature data were involved in the following analysis.

### 2.2 Subjects

All papers and thesis were reviewed carefully. Laurence-Moon syndrome (OMIM 245800) together with BBS were previously regarded as one disease (named Laurence-Moon-Biedl syndrome or Laurence-Moon-Bardet-Biedl syndrome). Now, Laurence-Moon syndrome has been regarded as another disease. Hence, we removed Laurence-Moon syndrome patients. A total of 61 papers (Xu, 1991; Zhang et al., 1999; Lin et al., 2000; Shao et al., 2000; Shen et al., 2001; Li and Pang, 2003; Tong and Yu, 2003; Wang, 2003; Zhang and Hu, 2004; Zhou and Wang, 2005; Lei et al., 2006; Shou et al., 2006; Yang et al., 2006; Zhang et al., 2007a; Zhang et al., 2007b; Chen et al., 2007; Ke and An, 2007; Lin et al., 2007; Lu and Wang, 2007; Li et al., 2008a; Li et al., 2008b; Chen et al., 2008; Liang and Zhou, 2008; Liu et al., 2008; Si et al., 2008; Ye et al., 2008; Zhang et al., 2009; Shou et al., 2011; Xiao et al., 2011; Zheng et al., 2011; Zheng et al., 2012; Cheng et al., 2013; Jiang, 2013; Li et al., 2013; Lin and Zhang, 2014; Wang et al., 2014; Xia et al., 2014; Yi et al., 2014; Huang et al., 2015; Lou et al., 2015; Qi et al., 2015; Wu and Zhang, 2015; Chen et al., 2017; Li et al., 2017; Ding et al., 2018; Lin et al., 2018; Qu et al., 2018; Shen et al., 2018; Wang et al., 2018; Qiang et al., 2019; Tao et al., 2019; Wu et al., 2019; Li et al., 2020; Xie et al., 2021a; Liu et al., 2021; Li et al., 2022a; Li and Hu, 2022; Lu et al., 2022; Shao and Dong, 2022; Shen et al., 2022; Wei et al., 2022) , 4 thesis in Chinese (Han, 2011; Xin, 2014; Xiu, 2018; Li, 2021), and 24 English papers (Wu, 1956; Wei et al., 1998; Hou, 2004; Yang et al., 2008; Li et al., 2014; Xing et al., 2014; Qi et al., 2017; Li et al., 2019; Shen et al., 2019; Dan et al., 2020; Tao et al., 2020; Huang et al., 2021; Jing et al., 2021; Meng et al., 2021; Tang et al., 2021; Zhang et al., 2021; Li et al., 2022b; Cai et al., 2022; Dong et al., 2022; Shao et al., 2022; Tang et al., 2022; Tao et al., 2022; Yan et al., 2022) in PubMed from China were enrolled. Among these 96 papers and thesis, patients repeated in 6 papers were merged (Lei et al., 2006; Shou et al., 2006; Zhang et al., 2007a; Zhang et al., 2007b; Shou et al., 2011; Shao et al., 2022). Finally, a total of 153 patients with BBS were enrolled in this study. The special field of journals, profession of authors, demographic information, clinical data, and genotype was collected, and compared with previous reports. The description of variants were revised according to the ACMG guidelines.

### 2.3 Diagnostic criteria

In children, overweight and obesity were defined according to “Body mass index growth curves for Chinese children and adolescents aged 0–18 years” (Li et al., 2009a). In adults, overweight was defined as a BMI between 24.0 and 27.99 kg/m^2^ while obesity was defined as a BMI ≥28.00 kg/m^2^.

In children, hypertension was defined according to the “updating blood pressure references for Chinese children aged 3–17 years” (Fan et al., 2017). In adults, hypertension was defined as a diastolic blood pressure >90 mmHg and/or a systolic blood pressure >140 mmHg.

Hyperglycemia included impaired fasting glucose (fasting glucose 5.6–6.9 mmol/L) and impaired glucose tolerance (7.8–11.0 mmol/L). Diabetes was defined as fasting glucose ≥7.0 mmol/L and/or random blood glucose ≥11.1 mmol/L.

Short stature was defined according to the “height and weight standardized growth chats for Chinese children and adolescents aged 0–18 years” (Li et al., 2009b).

### 2.4 Statistical analysis

Statistical analyses were conducted using SPSS software (version 22). The Pearson’s chi-square test and Fisher’s exact test were used to measure enumeration data between subgroups. Quantitative data with a normal distribution were expressed as the means ± SDs and analyzed by the independent *t*-test. Quantitative data with skewed distributions were expressed as medians (minimums-maximums). Differences were considered statistically significant at *p* < 0.05.

## 3 Results

### 3.1 Demographics

Among 153 patients (Supplementary Table S1), there were 87 males, 53 females, and 13 unknown (including fetuses) (Li et al., 2019; Qiang et al., 2019; Li et al., 2020; Jing et al., 2021; Cai et al., 2022; Dong et al., 2022; Tang et al., 2022; Yan et al., 2022). Among 124 patients with diagnostic age (also excluding 10 fetuses), their age ranged from 1.2 to 44 years old with a mean of 16.70 ± 9.90 years old. Only 10 (7.93%) were younger than 6 years old; and 69 (54.76%) were 7–18 years old, and 51 (40.48%) were older than 18 years. In our series, 58 patients (37.91%) had a family history of the BBS or similar disease, and the parents of 27 patients (17.65%) were consanguineous.

For the reporters, 80 patients (52.29%) were reported by ophthalmologists, following by pediatricians (24, 15.68%), endocrinologists (20, 13.07%), nephrologists (12, 7.84%), and obstetricians (10, 6.54%). These patients were reported from 23 provinces or regions and mostly from the Eastern region of China, including 34 (22.22%) from Beijing city, 21 (13.73%) from Guangdong province, 12 (7.84%) from Zhejiang province, Sichuan province, and Chongqing city, respectively. This was followed by Fujian province (8, 5.23%), Yunnan province (7, 4.58%), Xinjiang autonomous region (7, 4.58%), and Shanghai city (6, 3.92%).

### 3.2 Clinical characteristics

#### 3.2.1 Chief complaint

Among 91 patients with proven chief complaints, the most common were visual impairment (37, 38.14%) and obesity (20, 21.97%), followed by polydipsia and/or polyuria (12, 13.17%), abnormal renal image or function (18, 19.78%), polydactyly (12, 13.17%), intellectual disability (9, 9.89%), growth retardation (6, 6.59%), and abnormalities of the reproductive system (4, 4.40%). Malnutrition, anemia, convulsion, dyskinesia, fever and cough, weakness, hypertension, and hyperglycemia were also reported. Among 11 fetuses (Hou, 2004; Li et al., 2019; Qiang et al., 2019; Li et al., 2020; Jing et al., 2021; Cai et al., 2022; Dong et al., 2022; Yan et al., 2022), polydactyly and/or abnormal renal images were found by pregnancy examination, and hydrometrocolpos and vaginal atresia were reported in one fetus (Hou, 2004).

#### 3.2.2 Polydactyly

Among 153 patients, polydactyly was reported in 131 (85.62%), which was similar with Gnanasekaran et al. report (87/108, 80.55%; χ^2^ = 1.180, *p* = 0.277), but significantly higher than those in Beales et al. (1999) (75/109, 68.81%; χ^2^ = 10.707, *p* = 0.001) or Moore et al. (2005) (29/46, 63.04%; χ^2^ = 11.441, *p* = 0.001) reports. Of these, only 2 (1.31%) had postaxial 7-finger deformity, and the other 128 had postaxial 6-finger deformity. At least 97 patients (62.75%) involved fingers and 103 (67.32%) toes, and 85 (55.56%) involved both fingers and toes. Among 110 with detail about polydactyly, bimanual bipedal deformity was reported in 55 (50.00%) patients, with bimanual deformity only in 5 (4.54%), bipedal deformity only in 12 (10.91%), unimanual deformity only in 5 (4.54%), unipedal deformity in 4 (3.64%); unimanual bipedal deformity in 13 (11.82%), bimanual unipedal deformity in 6 (5.45%), and unimanual unipedal deformity 10 (9.09%), as shown in Table 1. Moreover, 25 patients (16.34%) had varying degrees of brachydactyly, 6 (5.45%) had syndactyly, and one had symptoms of thumb contracture. Most patients undergo surgery to remove an extra finger or toe for cosmetic reasons during childhood.

**TABLE 1:** Clinical features in the current series and previous reports (number, %).

	Current data	Gnanasekaran H. Indian (Gnanasekaran et al., 2023)	Mujahid S. United Kingdom (Mujahid et al., 2018)	Moore SJ. Canada (Moore et al., 2005)	Beales PL. United Kingdom (Beales et al., 1999)
Female/male	53/87	40/63	68/84	20/26	47/62
Age (year)	16.70 ± 9.90	3.8 ± 1.09 (onset age)	33.2 ± 11.8		9
Polydactyly	131/153 (85.62)	87 (80.55)		29/46 (63.04)	75/109 (68.81)
Four limbs	55/97 (55.56)				23/109 (21.10)
Visual problems	132/137 (96.35)				
Blind	25/132 (18.25)			42/46 (91.30)	
Onset age of blind	Approximately 20 years			18 (9–36)	
Retinitis pigmentosa	120/137 (87.59)	108 (100.00)			102/109 (93.58)
Overweight and obesity	124/132 (93.94)	77 (71.30)	101/131 (77.10)	45/45 (100)^a^	78/102 (76.47)
BMI (kg/m^2^)	29.08 ± 6.40		35.7 ± 8.0		31.5 ± 5.7
Raised ALT	7/153 (4.57)		34/127 (26.77)		
Dyslipidemia	21/153 (13.73)	7 (6.48, hypercholesterolemia)	71/130 (54.62)		
Hyperglycemia	21/153 (13.73)		28/109 (25.69)^b^		
Diabetes mellitus	9/153 (5.88)	8 (7.41)	25/109 (22.93)	22/46 (47.83)	7/109 (6.42)
Acanthosis nigricans	6/153 (3.92)				
Hypertension	32/153 (20.91)		67/100 (67.00)	31/46 (67.39)	7 (8)
Renal abnormalities	63/114 (55.26)	5 (4.63)		32/32 (100)	26/109 (23.85)
Renal cysts	25/114 (21.93)			23/32 (71.88)	6/57 (10.63)
Hypogenitalism	104/104 (79.81)	29 (26.85)	26/133 (19.55)		60/62 (96.77)^c^
Mental retardation	111/136 (81.62)	36 (33.33, learning disabilities) 34 (31.48, speech defects)		11/38 (28.94)	68/109 (62.39)
Hearing impairment	8/153 (5.23)	3 (2.78)			26/109 (23.85)
Epilepsy	3/153 (1.96)				4 (4%)
Dental anomalies	21/153 (13.73)	8 (7.41)			29/109 (26.61)
Congenital heart disease	8/153 (5.23)			3/46 (6.52)	8/109 (6.64)

^a^ One died after18 months.

^b^ Raised fasting plasma glucose.

^c^ For male. BMI, body mass index; ALT, alanine transaminase.

#### 3.2.3 Ocular characteristics

Among 137 patients with recorded ophthalmic examinations, 132 (96.35%) had visual problems (Table 1). The age of onset ranged from 1 to 17 years old with a median age of 5.0 (5.23 ± 3.65) years old in 53 patients with detail. Among these patients, 61 (44.53%) complained of nyctalopia/night blindness which is the initial manifestation of the disease in most people, and 25 (18.25%) blindness or almost blindness (only sense light, hand movement or count fingers) in their age of 20 s. The incidence of blindness was significantly lower than that of Moore et al. (2005) (42/46, 91.30%; χ^2^ = 97.823, *p* < 0.001) report. Retinitis pigmentosa was reported in 120 patients (87.59%). Moreover, restricted visual fields (15, 10.95%), nystagmus (22, 16.069%), strabismus (20, 14.59%), optic nerve atrophy (12, 8.76%), cataracts (12, 8.76%), and astigmatism (6, 4.38%) were also reported.

#### 3.2.4 Obesity

Among 132 patients with body mass index (BMI) or body shape, 11 (8.33%) were overweight and 113 (85.61%) were obese. Their BMI ranged from 11.94 to 48.89 kg/m^2^ with a mean BMI of 29.08 ± 6.40 kg/m^2^ among 102 patients. The incidence of overweight and obesity in our series (123/132, 93.18%) was higher than those in Gnanasekaran et al. (2023) (77/108, 71.30%; χ^2^ = 7.375, *p* = 0.007), Beales et al. (1999) (78/102, 76.47%; χ^2^ = 14.874, *p* < 0.001), and Mujahid et al. (2018) (101/131, 77.10%; χ^2^ = 15.084, *p* < 0.001) reports, but had no significant difference form and Moore et al. (2005) (45/45, 100%; *p* = 0.205) report. It was notable that hypertension was reported in 32 patients (20.91%). Moreover, hypertriglyceridemia was reported in 21 (13.73%) patients, with hyperglycemia in 21 (including 9 patients with diabetes mellitus), raised alanine transaminase in 7 (4.57%), fatty liver in 19 (12.42%, ultrasound report), and acanthosis nigricans in 6 (3.92%). Most incidences of these abnormalities were lower than those preorts by Mujahid et al. (2018) and Moore et al. (2005) reports, as shown in Table 1.

#### 3.2.5 Genitourinary system abnormalities

Among 114 patients with urinary data, 63 (55.26%) had varying degrees of renal abnormalities, including cysts in 25 (21.93%, including polycystic kidney in 4), small kidneys (renal atrophy or dysplasia) in 13 (11.40%), large kidneys in 9 (7.89%), and bladder diverticulum and renal calculus, in one patient, respectively. Recurrent urinary tract infections were reported in 2 patients. In this series, kidney dysfunction was found in 33 (21.57%) patients. The incidences of renal cysts was similar to that reported by Beales et al. (1999) (6/57, 10.63%; χ^2^ = 3.329, *p* = 0.068), but much lower than that in Moore et al. (2005) report (23/32, 71.88%; χ^2^ = 28.244, *p* < 0.001), and much higher than that in Gnanasekaran et al. (Farag and Teebi, 1989) report (5/108, 4.63%; χ^2^ = 66.916, *p* < 0.001), as shown in Table 1.

Of the 104 patients with reproductive records, 83 (79.81%) had varying degrees of developmental abnormalities, while 21 patients (20.19%) had no significant abnormalities. Most of the symptoms are hypogonadism and/or genital hypoplasia, such as micropenis, small testicles, cryptorchidism, hypospadias in males, or vulva and breast hypoplasia, uterine and ovary aplasia, and delayed and irregular menstruation in females. The abnormalities of the reproductive system were lower than those in Beales et al. (1999) report for males (60/62, 96.77%; χ^2^ = 9.368, *p* = 0.002), but significantly higher than those in Gnanasekaran et al. (Farag and Teebi, 1989) report (29/108, 26.85%; χ^2^ = 59.621, *p* < 0.001) and Mujahid et al. (2018) (10/68, 14.7%; χ^2^ = 70.169, *p* < 0.001) reports. Polycystic ovary syndrome, hydrometrocolpos and vaginal dysplasia, which were reported by Mujahid et al. (2018), were reported only in one patient, respectively (Hou, 2004; Meng et al., 2021). Notably, precocious puberty was reported in 2 patients (including central precocious puberty in one patient) (Li et al., 2022a; Li et al., 2022b).

#### 3.2.6 Nervous system abnormalities

Mental retardation (including learning difficulties and speech delay) was reported in 111 (81.62%) of 136 patients, which was manifested by an inability to complete school or even to take care of themselves (Table 1). This value was significantly higher than that in reports from Beales et al. (1999) (68/109, 62.39%; χ^2^ = 11.371, *p* = 0.001) or Moore et al. (2005) (11/38, 28.94%; χ^2^ = 39.322, *p* < 0.001) reports. However, only 13 patients (9.55%) had detailed records of intelligence test results. Motor development delay was alos reported in 4 patients, ataxia/poor coordination in 3 (Huang et al., 2021), and epilepsy was reported in 3 (Wang et al., 2014; Meng et al., 2021).

#### 3.2.7 Other anomalies

Among 91 patients with height and short stature data, 56 (61.54%) were short stature (9 patients ranged from −1 SD to −2 SD, 34 less than −2 SD, 12 without height detail) and 35 (38.46%) were at the normal height for their age. The height of 11 male adults (≥18 years) ranged from 1.45 to 1.75 m with a mean height of 160.91 ± 10.57 m while that of 16 female adults (≥17 years) ranged from 1.36 to 1.56 m with 149.34 ± 8.68 m. Dental dysplasia was noted in 21 patients (13.73%), with hearing impairment in 8 (5.23%), congenital heart disease in 8 (including 4 atrial defects, 2 ventricular defect), osteoarthritis-like changes in 11 (7.19%), hemangioma in 3 (1.96%), short neck in 8 (5.23%), hypothyroidism in 3, Hirschsprung disease, anal stenosis, and anal atresia in one patient, respectively (Table 1).

In this series, 11 fetuses were diagnosed prenatally from 2019 to 2022 years (Li et al., 2019; Qiang et al., 2019; Li et al., 2020; Jing et al., 2021; Cai et al., 2022; Dong et al., 2022; Yan et al., 2022) except for one patient reported in 2004 (Hou, 2004). All fetuses presented abnormal kidney images and/or bilateral enlarged hyperechogenic kidneys that implied renal cysts, and one presented hydrometrocolpos and postaxial polydactyly with vaginal atresia (Hou, 2004). Oligohydramnios was noted in one, and ventricular defect combined with a single atrium, persistent left superior vena cava and ascites were noted in one patient.

### 3.3 Genotypes and phenotypes

#### 3.3.1 Genotypes

**FIGURE 1:**
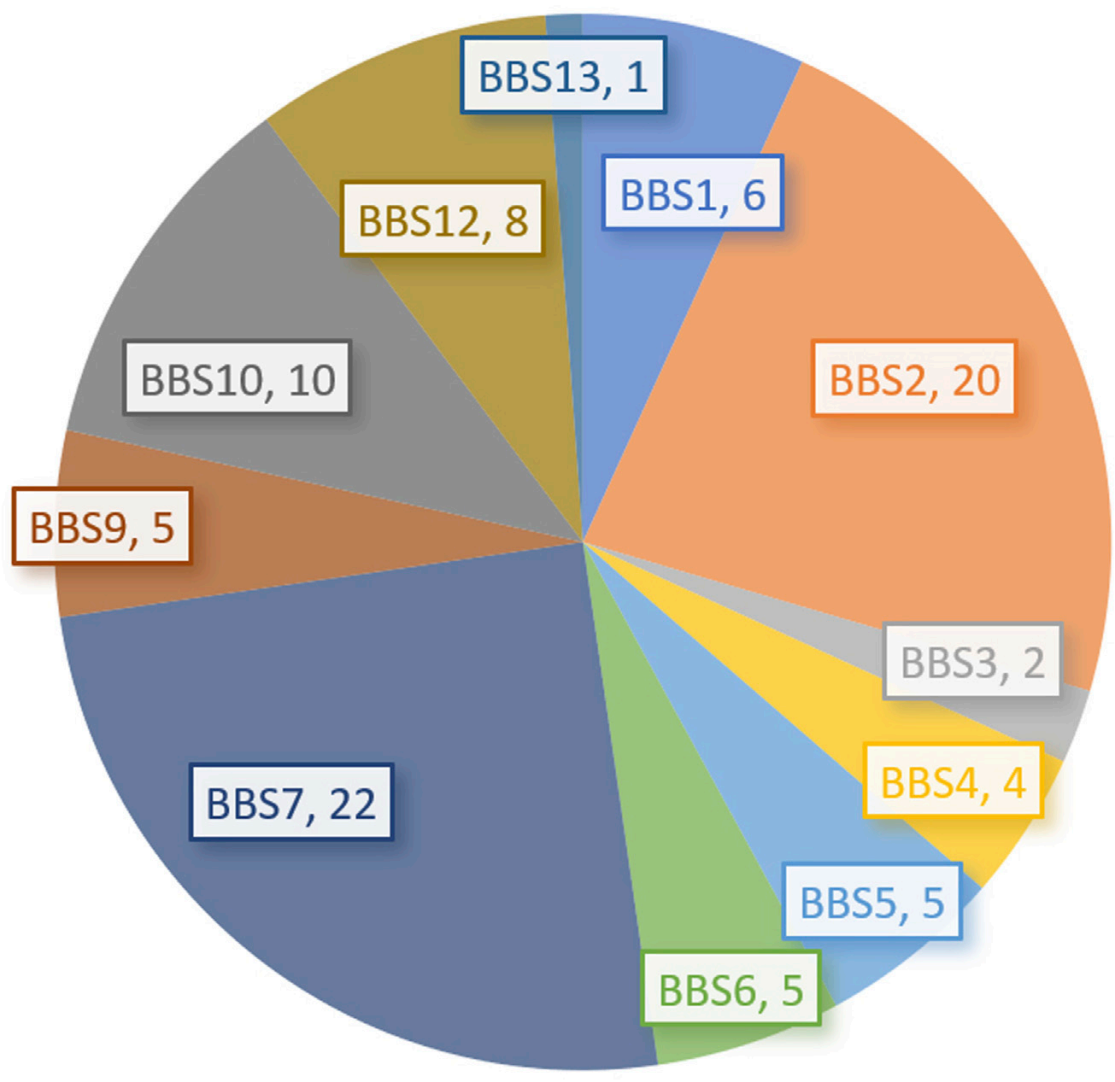
The genotypes of *BBS* in the current series (number).

Among 153 patients, genetic analysis was performed in only 90 patients (58.82%), including one negative finding (Tao et al., 2020). Except for 3 patients who were analyzed using linkage analysis (including 2 *BBS7* and one *BBS5*) in 2007 and 2008 (Chen et al., 2007; Liu et al., 2008), other 86 patients with genetic analysis were reported. Most (77, 89.53%) were reported in the last 6 years (from 2017 years). The most common genotypes were *BBS7* (22 patients, 24.71%), *BBS2* (20, 22.73%), and *BBS10* (10, 11.24%). These were followed by *BBS12* (8, 8.99%), *BBS1* (6, 6.74%), *BBS5* (5, 5.62%), *BBS6/*MKKS (5, 5.62%), *BBS9/PTHB1* (5, 5.62%), and *BBS4* (4, 4.49%), as shown in Table 2 and Figure 1. The proportions of *BBS2*, *BBS4*, *BBS7*, and *BBS9/PTHB1* were higher while the proportion of *BBS1* was lower in our Chinese series than those in most reports outside China (Moore et al., 2005; Forsythe and Beales, 2013; Mujahid et al., 2018; Guardiola et al., 2021). Moreover, the affected proteins of 62 patients (69.66%) were involved in the assembly of the BBSome complex while 23 (25.84%) were involved in the chaperone-like protein complexes. Among 81 patients with detailed variants, 42 (51.85%) were compound heterozygous variants, 38 (46.91%) were homozygous (including one uniparental disomy, and one perhaps uniparental disomy), and one was a heterozygous variant with a topical typical phenotype. Among 11 fetuses, 10 patients were reported in the last 4 years except for one reported in 2004 years (Hou, 2004). There were 6 patients (54.55%) with *BBS7* (Li et al., 2019; Li et al., 2020; Jing et al., 2021), 3 (27.27%) with *BBS1* (Qiang et al., 2019; Cai et al., 2022; Yan et al., 2022), one with *BBS6/*MKKS (Hou, 2004), and one with *BBS10* (Dong et al., 2022).

**TABLE 2:** The genotypes in the current series and several previous reports (number, %).

	Current data	Gnanasekaran H. Indian (Gnanasekaran et al., 2023)	Guardiola G. United States (Guardiola et al., 2021)	Mujahid S. United Kingdom (Mujahid et al., 2018)	Moore SJ, Canada (Moore et al., 2005)^a^	Forsythe E, review (Forsythe and Beales, 2013)
Total	89	76	27	152	40	
*BBS1*	6 (6.74)	7 (9.21)	23 (77.78)	71 (46.71)	8 (20.00)	23%
*BBS2*	20 (22.47)	6 (7.89)	0	17 (11.18)	1 (2.50)	8%
*BBS3/ARL6*	2 (2.25)	7 (9.21)	0	0	5 (12.50)	0
*BBS4*	4 (4.49)	3 (3.95)	0	0	0	2%
*BBS5*	5 (5.62)	6 (7.89)	0	0	5 (12.50)	0.4%
*BBS6/MKKS*	5 (5.62)	5 (6.58)	0	3	15 (37.50)	6%
*BBS7*	22 (24.72)	5 (6.58)	4 (17.39)	0	0	2%
*BBS8/TTC8*	0	2 (2.63)	0	1 (0.66)	0	1%
*BBS9/PTHB1*	5 (5.62)	3 (3.95)	0	1 (0.66)		6%
*BBS10*	10 (11.24)	19 (25.00)	0	20 (13.16)		20%
*BBS11*		1 (1.32)				
*BBS12*	8 (8.99)	10 (13.16)	0	8 (5.26)		5%
*BBS13/MKS1*	1 (1.12)		0	0		4.5%
*BBS16/SDCCAG8*	1 (1.12)					
*BBS17/LZTFL1*		2 (2.63)				
Unknown				30 (19.74)	5 (12.50)	

^a^ Only *BBS1* to *BBS8/*TTC8 were analyzed.

Except for 2 cases analyzed by linkage analysis without detailed data, 19 *BBS7* patients from 13 families were reported (Chen et al., 2007; Yang et al., 2008; Li et al., 2017; Xiu, 2018; Li et al., 2019; Shen et al., 2019; Tao et al., 2019; Li et al., 2020; Tao et al., 2020; Jing et al., 2021; Meng et al., 2021). Homozygous variants were noted in 10 patients (50.00%) and 10 (50.00%) compound heterozygous variants were noted in our series. Unlike a report from the United States (Guardiola et al., 2021), which reported hot spot variants in *BBS1* and *BBS7*, at least 11 variants of *BBS7* were reported in this series. The most common variant was the c.1002delT (p. Asn335Ilefs*47) variant, which was found in 7 patients (36.84%) from 5 families, including 2 homozygotes. This was followed by homozygous variant c.389_390delAC (p. Asn130Thrfs*3), which was reported in 5 patients from one family (Shen et al., 2019). The heterozygous variants c.288_289delAG (p. Gly97Lysfs*7) and p.Gly97Lysfs*7 were reported in 4 patients (15.79%) from 4 families, and c.728C>A (p. Cys243Tyr) and p. Cys243Tyr were reported in 4 patients (10.51%) from 4 families. The homozygous variant c.1666A>G (p. Ser556Arg) was reported in 2 patients from one family (Yang et al., 2008). Other variants included c.338C>A (p. Ala113Asp), c.497C>A (p. Ala166Asp), c.718G>A (p. Gly240Ser), c.849+1G>C, c.1395T>A (p. Tyr465*), and p. Ser117Pro. Moreover, the 4q26q27 microdeletion including *BBS7* was reported in 2 fetuses from one family.

Except for one case without detailed data of variant, 19 *BBS2* patients from 12 families were reported (Xing et al., 2014; Chen et al., 2017; Li et al., 2017; Ding et al., 2018; Dan et al., 2020; Huang et al., 2021; Meng et al., 2021; Tang et al., 2022; Tao et al., 2022; Wei et al., 2022), including 12 (63.16%) compound heterozygous variants and 7 (36.84%) homozygous variants. Unlike report from the United States (Guardiola et al., 2021), which reported hot spot variants in *BBS1* and *BBS7*, a total of 15 variants were reported in our series. The most common was the c.534+1G>T splicing variant in 9 patients (47.37%) from 5 families, including 2 homozygotes. It was notable that one patient with a homozygous c.534+1G>T splicing variant was associated with the paternal uniparental disomy (Wei et al., 2022). The heterozygous variant c.235T>G (p. Thr79Pro) were found in 4 patients (21.05%) from the same family. A c.563delT (p. Ile188Thrfs*13) variant was found in 3 patients (15.79%) from 3 families, including one homozygous. The homozygous variant c.79A>C (p. Thr27Pro) was found in 2 patients (10.53%) from one family. The heterozygous variants c.1278A>G (p. Glu426Glu) and c.2059C+1G>C splicing variant were reported in 2 patients from one and 2 families, respectively. Other variants included c.646C>T (p. Arg216*), c.944G>A (p. Arg315Gln), c.1015C>T (p. Arg339*), c.1148_1149dupTC (p. His384Serfs*34), c.1206dupA (p. Arg403fs*216), c.1237C>T (p. Arg413*), c.1438C>T (p.Arg480*), p. Tyr229*, and p. Arg703*.

Among 10 *BBS10* patients (Lin et al., 2018; Wang et al., 2018; Tao et al., 2020; Liu et al., 2021; Li et al., 2022a; Li et al., 2022b; Dong et al., 2022; Lu et al., 2022; Tao et al., 2022; Yan et al., 2022), 7 had compound heterozygous variants and 3 had homozygous variants. A total of 11 variants were also reported. The most common variant was c.539G>A (p. Gly180Glu), which was found in 4 patients (40.0%) in 4 families, including one homozygous. The heterozygous variant c.1391C>G (p. Ser464*) was found in 3 patients (30.0%) in 3 families, and the heterozygous variant c.602G>A (p. Cys201Tyr) was found in 2 patients (20.0%) in 2 families. Other variants included c.184C>T (p. His62Tyr), c.378G>A (p. Trp126*), c.445_446insC (p. Leu149Pfs*3), c.784_785delGA (p. Glu262Asnfs*41), c.891_897delinsTTTGT (p. Met298Leufs*5), 1063C>T (p. Gln355*), c.1812dupT (p. Asn605*), and c.1949delA (p. His650Profs*12). It was notable that 5 (45.45%) of 11 variants were Indel variants.

#### 3.3.2 Genotype-phenotype relationship

We compared the phenotypes among *BBS2*, *BBS7*, and *BBS10* patients. It was noted that *BBS7* had higher penetrance. *BBS2* had higher hearing impairment and lower renal abnormality penetrance. Moreover, *BBS2* had lower mental retardation and polydactyly penetrance with marginal differneces. *BBS10* had higher penetrance except for the lower renal abnormality as well (Table 3).

**TABLE 3:** Genotype and phenotype relationship (number, %).

	*BBS7*	*BBS2*	*BBS10*	χ^2^	*p*-value
Female/Male/unknown	4/15/3^a^	7/12/1	5/4/1^b^	3.349	0.187
Ocular problem	17/17 (100.00)	20/20 (100.00)	8/9 (88.89)	4.202	0.122
Retinitis pigmentosa	16/16 (100.00)	19/20 (95.00)	8/8 (100.00)	1.228	0.541
Renal abnormality	14/18 (77.78)	3/15 (20.00)	3/8 (37.50)^c^	11.438	0.003
Reproductive system	14/16 (87.50)	11/16 (68.75)	6/8 (75.00)^d^	1.649	0.439
Polydactyly	21/21 (100.00)	15/20 (75.00)	8/10 (80.00)	5.820	0.054
Overweight and obesity	17/17 (100.00)	18/19 (94.74)	9/9 (100.00)	1.400	0.497
Mental retardation	15/17 (88.24)	9/17 (52.94)	7/9 (77.78)	5.446	0.066
Hypertension	3/19 (15.79)	9/20 (45.00)	2/9 (22.22)	4.283	0.117
Hearing impairment	0/19	4/20 (20.00)	0/9	6.109	0.047

^a^ 2 fetuses.

^b^ One fetus.

^c^ One patient with renal calculus.

^d^ One patient with central precocious puberty.

## 4 Discussion

BBS is a main cause of syndromic forms of obesity although it is a rare disease. According to the prevalence (about 1/125,000∼1/160,000) reported (Moore et al., 2005; Tsang et al., 2018), these are currently about 10,000 patients in China (about 1.4 billion people) now. However, fewer than 150 patients have been reported to date. Moreover, most cases were reported from the Eastern region. This finding implied that misdiagnosis or miss diagnosis of BBS was common in China, especially in the Midwest region, although it may also be because of the non-register system of BBS in China.

Although it was an autosomal recessive disease, we noted that BBS was predominantly male with the male to female ratio of 1.64:1 (87/53). This result was similar to that in most other studies by Beales et al. (1999) (1.3:1) and Klein and Ammann (1969) (1.4:1), but the reason is still unclear. Whether this is associated with the fact that dysplasia of the vagina, uterus, and ovaries may be more apt to be ignored than cryptorchidism and micropenis needs further investigation. Although the age at diagnosis may be younger than those in other studies from Canada in 2005 (Moore et al., 2005) and the United Kingdom in 2018 (Mujahid et al., 2018), the mean age of these Chinese patients was 16.7 years old. This implies that the delayed diagnosis is still common. It was also notable that while only 15.68% of patients were reported by pediatricians, most (52.29%) were reported by ophthalmologists. In fact, polydactyly, a main feature and early clue of BBS, was noted in 85.62% of patients in this series. Hence, in addition to visual impairment, obesity, and genitourinary system abnormalities, BBS should be considered in infants (even fetuses) with polydactyly by pediatricians and pediatric surgeons.

The phenotype of BBS is heterogeneous. In our Chinese series, the incidences of visual impairment, polydactyly, obesity, genital anomalies, renal anomalies, and mental retardation were higher than those in most previous reports, although the age ass not older than those in previous reports (Beales et al., 1999; Moore et al., 2005; Mujahid et al., 2018). This implies that some atypical patients with few main clinical features may be misdiagnosis in China. It was notable that the incidence of some non-main clinical features (e.g., hearing loss, dental anomalies, anosmia/hyposmia, hydrometrocolpos, vaginal dysplasia, polycystic ovary syndrome in females) and complications (e.g., hypertriglyceridemia, hyperglycemia, diabetes millitus, and hypertension) were significantly lower than those in previous reports (Beales et al., 1999; Moore et al., 2005; Mujahid et al., 2018). These results suggest that careful evaluate of various deformities and congratulations on defects is needed for Chinese patients.

To date, 28 genes have been reported to be associated with BBS phenotypes (Niederlova et al., 2019; Gnanasekaran et al., 2023; Khan et al., 2023). Unlike Caucasian patients with higher proportions of *BBS1* and *BBS10* (Niederlova et al., 2019; Melluso et al., 2023), we noted that *BBS7* was the prominent genotype, followed by *BBS2*, *BBS10*, *BBS12*, and *BBS1* in these Chinese seies. This difference may imply the different genotype prominence in different geographic areas. This may also be associated with the fact that poor clinical diagnosis as *BBS1* patients tend to have a milder pattern of disease (Niederlova et al., 2019; Guardiola et al., 2021). The analysis for the relationship between genotype and phenotype is difficult, as this rare disease. The BBSome chaperone-like protein is involved in the early synthesis of BBSome, so patients with *BBS6/*MKKS, *BBS10*, and *BBS12* have more severe symptoms, especially *BBS10* (Forsythe et al., 2017; Niederlova et al., 2019). Moreover, patients with *BBS2* were reported to have severe symptoms (Florea et al., 2021). The proportion of renal abnormalities in patients with *BBS7* types is relatively high (>60%) and relatively lower in *BBS2* and *BBS10* types (Niederlova et al., 2019; Florea et al., 2021). Patients with *BBS2* and *BBS4* types have a higher proportion of polydactyly (Niederlova et al., 2019), and *BBS10* and *BBS12* types are apt to obese (Forsythe and Beales, 2013; Dai et al., 2022; Melluso et al., 2023). We noted that *BBS7* had higher penetrance. *BBS2* had higher hearing impairment and lower renal abnormality penetrance. *BBS10* had higher penetrance except for the lower enal abnormality. It was notable that hearing impairment was reported in *BBS2*, but not in *BBS7* and *BBS10*. The relationship between genotype and phenotype must be observed with a larger sample size.

The diagnosis of BBS is some difficult due to the heterogeneity of phenotypes. Beales et al. (1999) summarized and amended that BBS clinical diagnosis should meet 4 major symptoms, or 3 major symptoms and 2 secondary symptoms. Fortunately, genetic diagnosis has been improved in recent years in China, and 89 patients had comfirmed diagnosis and genotyping by sequencing in recent years. Unlike reports from the United States (Guardiola et al., 2021), the variant sites are more dispersed in our Chinese series. As most patients have point variants, sequencing, but not karyotype and array comparative genomic hybridization (aCGH), is suggested as the first line genetic analysis. Moreover, we also used next-generation sequencing for newborn screening in China, which may improve the early diagnosis for some rare genetic diseases (Tong et al., 2022).

The diagnosis of BBS based on prenatal findings is still some difficult, as it cannot identify visual impairment, learning difficulties, or obesity *in utero*. Fortunately, prenatal diagnosis war comfirmed by sequencing for 10 fetuses after renal anomalies and/or polydactyly found by ultrasound in the past 4 years. Hence, in fetuses with genitourinary abnormalities, polydactyly, and/or hydrometrocolpos, BBS should be considered. Further careful evaluation for various deformities and genomic sequencing should be suggested.

Genetic counseling for the family of the proband is needed. As BBS is an autosomal recessive disease, consanguineous marriage should be avoided. The consanguinity rate in our series was 17.65%, which was lower than that in other studies by Beales et al. (1999) (39%) and Klein and Ammann (1969) (48%), and may be associated with more dispersed variant sites in our series. However, this reflected the lack of awareness of the dangers of consanguineous marriage in these Chinese families.

There is still no specific treatment for BBS. Congenital structural abnormalities (e.g., digestive tract abnormalities), obesity and metabolic syndrome, chronic kidney disease, and retinitis pigmentosa are the main influencing factors for the quality of life and longevity of BBS patients. Congenital structural abnormality correction, diet and lifestyle interventions to prevent obesity and metabolic syndrome, relieve chronic kidney disease, and slow down the retinitis pigmentosa progression are important for BBS patients. Drugs (e.g., setmelanotide) for the obesity (Tauber, 2022; Forsythe et al., 2023), and gene therapy for retinitis pigmentosa are in clinical trails (Xie et al., 2021b; Hsu et al., 2023).

There were several limitations. First, some patients were reported having “Laurence-Moon-Biedl syndrome” or “Laurence-Moon-Bardet-Biedl syndrome”. It may not be accurate to exclude some patients without genetic analysis data. Second, as a review analysis, some clinical data of some patients were not provided. Moreover, genetic analysis was not performed in all patients, and most patients reported before 2017 years. Hence, the genotype-phenotype relationship analysis may not be accurate.

In summary, misdiagnosis or miss diagnosis of BBS may be common in China. In patients with polydactyly, visual impairment, obesity, renal abnormalities, hypogonadism, and mental retardation, or in fetuses with polydactyly and/or renal abnormalities, BBS should be considered in the differential diagnosis. Other deformities should be evaluated carefully and genetic analysis should be performed as early as possible.

## Data Availability

The original contributions presented in the study are included in the article/Supplementary Material, further inquiries can be directed to the corresponding author.
